# The adhesion molecule CD44v6 is associated with a high risk for local recurrence in adult soft tissue sarcomas

**DOI:** 10.1054/bjoc.2000.1590

**Published:** 2001-01

**Authors:** S Maula, R L Huuhtanen, C P Blomqvist, T A Wiklund, P Laurila, R Ristamäki

**Affiliations:** The National Public Health Institute, MediCity Research Laboratory, Turku University, Tykistökatu 6 A, Turku, FIN-20520, Finland; Department of Oncology, Helsinki University Central Hospital, HYKS, FIN-00029, Finland; Department of Oncology, Uppsala University, Uppsala, S-75185, Sweden; Department of Pathology, Helsinki University Central Hospital, HYKS, FIN-00029, Finland; Department of Oncology, Turku University Central Hospital, Kiinamyllynkatu 4–6, Turku, FIN-20520, Finland

**Keywords:** soft-tissue sarcoma, CD44, CD44v6, prognosis, local recurrence

## Abstract

In many malignant diseases the expression levels of CD44 and its splice variant v6 (CD44v6) have been associated with the prognosis. The purpose of this study was to investigate the clinical significance of CD44 in adult soft tissue sarcomas (STS). 133 STS patients with a limb or superficial trunk tumour treated at the Helsinki University Central Hospital in 1987–1993 with a median follow-up time of 68 months were included in this study. The expression of CD44 and CD44v6 was determined immunohistochemically on paraffin-embedded tumour samples. 95% of the tumours expressed CD44 and CD44v6 was detected in 57%. Strong CD44 expression was associated with low grade (*P* = 0.04) and small tumour size (*P* = 0.02). In diploid tumours the CD44 expression was correlated with low S-phase fraction (*P* = 0.001). High expression of both, CD44 in general as well as that of CD44v6, predicted a higher risk for local recurrence (CD44: *P* = 0.01 and CD44v6: *P* = 0.05). Low CD44v6 content of the primary tumour correlated with poor survival (*P* = 0.02). Determining the expression of CD44 or CD44v6 in a primary STS could be a valuable tool for selecting the group of patients who might benefit from intensified local tumour treatment. © 2001 Cancer Research Campaign http://www.bjcancer.com


*British Journal of Cancer* (2001) **84**(2), 244–252
© 2001 Cancer Research Campaign
doi: 10.1054/ bjoc.2000.1590, available online at http://www.idealibrary.com on IDEAL®

http://www.bjcancer.com

### The adhesion molecule CD44v6 is associated with a high risk for local recurrence in adult soft tissue sarcomas

**S Maula[1], RL Huuhtanen[2], CP Blomqvist[3], TA Wiklund[2], P Laurila[4] and R Ristamäki[5]**

[1] The National Public Health Institute, MediCity Research Laboratory, Turku University, Tykistökatu 6 A, FIN-20520 Turku, Finland;[2] Helsinki University Central Hospital, Department of Oncology, FIN-00029 HYKS, Finland; [3] Department of Oncology, Uppsala University, S-75185 Uppsala, Sweden; [4]Helsinki University Central Hospital, Department of Pathology, FIN-00029 HYKS, Finland; [5]Turku University Central Hospital, Department of Oncology, Kiinamyllynkatu 4–6, FIN-20520 Turku, Finland

**Summary** In many malignant diseases the expression levels of CD44 and its splice variant v6 (CD44v6) have been associated with the prognosis. The purpose of this study was to investigate the clinical significance of CD44 in adult soft tissue sarcomas (STS). 133 STS patients with a limb or superficial trunk tumour treated at the Helsinki University Central Hospital in 1987–1993 with a median follow-up time of 68 months were included in this study. The expression of CD44 and CD44v6 was determined immunohistochemically on paraffin-embedded tumour samples. 95% of the tumours expressed CD44 and CD44v6 was detected in 57%. Strong CD44 expression was associated with low grade ($P$ = 0.04) and small tumour size ($P$ = 0.02). In diploid tumours the CD44 expression was correlated with low S-phase fraction ($P$ = 0.001). High expression of both, CD44 in general as well as that of CD44v6, predicted a higher risk for local recurrence (CD44: $P$ = 0.01 and CD44v6: $P$ = 0.05). Low CD44v6 content of the primary tumour correlated with poor survival ($P$ = 0.02). Determining the expression of CD44 or CD44v6 in a primary STS could be a valuable tool for selecting the group of patients who might benefit from intensified local tumour treatment. © 2001 Cancer Research Campaign http://www.bjcancer.com

**Keywords**: soft-tissue sarcoma; CD44; CD44v6; prognosis; local recurrence

Soft tissue sarcomas (STS) form a heterogeneous group of tumours both in pathological and clinical sense thus leading in therapeutic difficulties (Dirix and Van Oosterom, 1995). Favourable outcome of the disease not only requires early diagnosis but also rather aggressive first line treatment combining extensive surgery, radiotherapy and in selected cases also chemotherapy. Currently, prognosis estimations are based on the histological grade, size and the depth of the tumour (Collin et al, 1987; Rööser et al, 1987; Alvegård et al, 1989). Meanwhile, new prognostic factors are actively being searched for, mainly in the field of tumour biology. Cell proliferation markers such as flow cytometrically defined S-phase fraction (SPF) describing the proportion of dividing cells (Huuhtanen et al, 1996; Collin et al, 1997) as well as the expression of cell cycle antigens Ki-67 and PCNA (Choong at al, 1995) seem to have prognostic value in STS. Overexpression of certain inactive mutated forms of the tumour suppressor gene *p53* has also been shown to predict unfavourable outcome in STS (Taubert et al, 1996). However, further studies are needed to find out the most useful prognostic parameters for the future clinical practice.

Cell–cell and cell–matrix interactions are essential for normal growth and differentiation of cells as well as for tumour development and invasion. A variety of adhesion molecules participate in these interactions and CD44 is one of them. CD44 comprises a family of hyaluronic acid binding cell surface glycoproteins encoded by a cDNA with 20 exons. 10 of them, coding for the extracellular domains, can be alternatively spliced thereby producing a large number of variant molecules (CD44v). Further diversity can be accomplished by post-translational modifications, mainly glycosylation. Both alternative splicing and glycosylation influence the function of a particular CD44 isoform (reviewed in Naor et al, 1997) by affecting the ligand-binding properties of the molecule. Overall, CD44 molecules are involved in various physiological functions. They participate in lymphocyte homing (Jalkanen et al, 1986) and mediate cell adhesion to several extracellular matrix components (Miyake et al, 1990a). They have a role in lymphohaematopoiesis (Miyake et al, 1990b) in homotypic cell adhesion (Belitsos et al, 1990), in T-cell activation and adhesion (Denning et al, 1984), in cytokine release (Webb et al, 1990) and lateral movement of cells (Jacobson et al, 1994).

The standard form of CD44 is widely expressed in several tissue types while distribution of the variant forms is mainly restricted to different epithelia (Mackay et al, 1994). The significance of several CD44 isoforms, including CD44v6, in the biology of malignant diseases has been shown in a number of animal and human studies (Günthert et al, 1991; Naor et al, 1997). Neo-expression or upregulation of a certain CD44 splice variant has been associated with poor survival rate in many malignant diseases, whereas in others unfavourable outcome occurs when the tumour looses its CD44 expression (Naor et al, 1997).

There is not much data of adhesion molecule expression and their significance in STS as a group. We chose to retrospectively investigate the expression of CD44 as such and the expression of CD44v6 in STS, since many times they have been shown to have a role in tumour biology and to reflect the clinical behaviour of a

*Received 27 April 2000*
*Revised 12 September 2000*
*Accepted 18 October 2000*

*Correspondence to:* S Maula malignant disease. A standard immunohistochemical analysis of the expression of these molecules was performed to visualize their distribution in primary soft tissue sarcomas. The expression levels were correlated with several widely used clinicopathological parameters and the prognosis.

### MATERIALS AND METHODS

### Patients

Originally 155 adult patients with a limb or superficial trunk tumour treated at the Helsinki University Central Hospital (HUCH) in January 1987–May 1993 were included in this study. In each case the tumour diagnosis was based on histopathological examination. Well-preserved paraffin blocks of the primary tumour were available in 133 cases and the samples were all taken before the beginning of any treatment. The median age of the patients was 54 years (range from 18 to 89 years) at the time of diagnosis. The female to male ratio was 5:4 (74 and 59 patients, respectively). The treatment of these patients was based on the guidelines set by the Helsinki STS group. Surgery alone with wide or compartmental margin as well as surgery with marginal margin combined with postoperative radiotherapy were considered as adequate treatment protocols for the primary neoplastic lesion based on the results from a previous study (Wiklund et al, 1996). Three patients with extraskeletal Ewing's sarcoma received preoperative chemotherapy as part of primary treatment and another three patients had chemotherapy after the operation. All the patients were regularly followed-up at the outpatient department. The median follow-up time of living patients was 68 months (range from 9 to 453 months). During the follow-up period 43 patients (32%) developed a local tumour recurrence, and 50 patients (38%) received metastases. 53 patients (40%) have died since, 37 (28%) of whom from STS. Detailed pre-treatment characteristics are presented in Table 1.

The Helsinki STS team is part of the larger Scandinavian Sarcoma Group that applies a four-grade grading system for histological malignancy. The grading is based on histology and histopathological parameters such as necrosis, vascular invasion, cellularity, mitotic activity and nuclear pleomorphism. All the histological diagnoses were reconfirmed by a pathologist specialized in sarcomas (PL). Measurements of cell proliferation rate as S-phase fraction determined by DNA flow cytometry, and the nuclear antigen Ki-67 expression detected by immunohistochemistry were performed earlier (Huuhtanen et al, 1996; Huuhtanen et al, 1999). The median SPF for all the tumours was 7.3% (range from 0.0 to 38.2%), for diploid tumours it was 4.05% (range from 0.80 to 21.9%) and for non-diploid tumours it was 12.75% (range from 0 to 38.2%), respectively (Huuhtanen et al, 1996).

**Table 1** Pretreatment characteristics

| Parameter | *n* | % |
|---|---|---|
| Size | | |
| < 5 cm | 56 | 42 |
| ≥ 5 cm | 74 | 56 |
| Unknown | 3 | 2 |
| Grade | | |
| I | 8 | 6 |
| II | 24 | 19 |
| III | 50 | 40 |
| IV | 43 | 35 |
| Location | | |
| Trunk | 32 | 21 |
| Limb | 123 | 79 |
| Site | | |
| Only cutaneous/subcutaneous | 90 | 58 |
| Deep | 63 | 41 |
| Unknown | 2 | 1 |
| Ploidy | | |
| Diploid | 68 | 51 |
| Aneuploid | 65 | 49 |
| Histological type | | |
| Malignant fibrous histiocytoma | 37 | 28 |
| Liposarcoma | 15 | 12 |
| Leiomyosarcoma | 14 | 12 |
| Synovial sarcoma | 8 | 6 |
| Fibrosarcoma | 8 | 6 |
| Dermatofibrosarcoma | 8 | 6 |
| Extraskeletal Ewing's sarcoma | 7 | 5 |
| Malignant schwannoma | 4 | 3 |
| Extraskeletal osteosarcoma | 2 | 1 |
| Epitheloid sarcoma | 2 | 1 |
| Angiosarcoma | 2 | 1 |
| Other sarcomas[a] | 7 | 5 |
| Unclassified sarcoma | 19 | 14 |

[a]Other sarcomas: myxofibrosarcoma, extraskeletal myxoid chondrosarcoma, clear cell sarcoma, rhabdomyosarcoma, alveolar rhabdomyosarcoma, stromal sarcoma and malignant hemangiopericytoma.

### Monoclonal antibodies

Monoclonal antibody (mAb) Hermes-3 recognizes an epitope in the constant part of CD44 (Jalkanen et al, 1987). mAb 20E6, rat IgG1 against human CD44v6, was produced by immunizing Spraque-Dawley rats with purified CDv6-v10 antigen in incomplete Freund's adjuvant by an injection into the footpads 3 times at 1-week intervals. Popliteal lymph node lymphocytes were isolated and fused with NS-1 myeloma cells according to a standard procedure. The positive hybridoma, 20E6, was subcloned twice.

Namalwa cells transfected either with CD44v6-10, CD44v7–10 or with the standard CD44 only were used for testing the hybridoma supernatants by a single-colour indirect immunofluorescence staining. Shortly, stable CD44 transfectants were incubated with hybridoma supernatants for 20 minutes, followed by secondary antibody that was a FITC-conjugated goat antirat IgG (Sigma). Analyses were performed by FACScan cytometer (Becton Dickinson, Mountain View, CA). mAb 20E6 was shown to recognize CD44 isoform containing the exon v6 domain (Figure 1). A more detailed description of the preparation of the Namalwa transfectants is presented elsewhere (Aho et al, 1997). Tonsil sections were also stained immunohistochemically with 20E6 and it was found to function well on both frozen and paraffin-embedded samples.

As a negative control we used 3G6, a mAb against avian T-cells. All these antibodies were used either as supernatants or ammonium sulphate precipitated concentrates. To stain the epithelial components of the synovial sarcomas we used a commercial anti-cytokeratin mAb AE1/AE3 (Boehringer-Mannheim, Mannheim, Germany).

### Immunohistochemical techniques

A standard immunoperoxidase staining procedure was carried out to detect the expression of the molecules studied. Briefly, 5 μm sections were cut and fixed on glass by incubating at 37°C overnight. Dewaxing was done in xylene. To rehydrate the

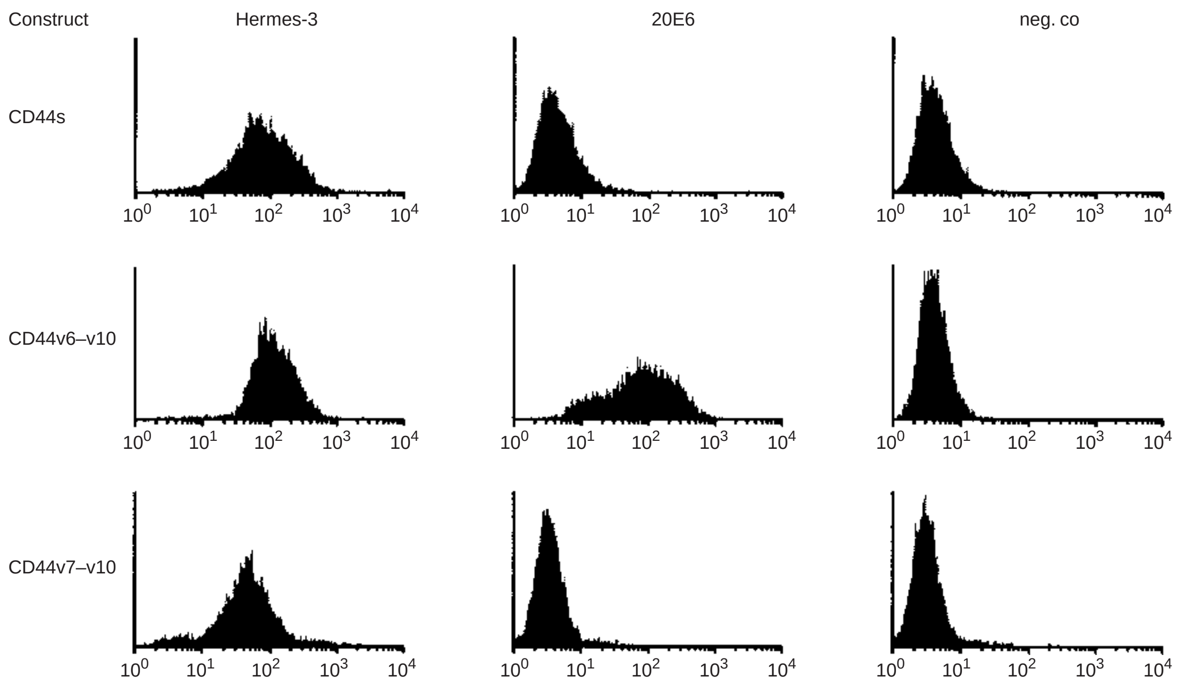

**Figure 1** Specificity of mAb 20E6. Hermes-3 recognizes all CD44 isoforms. 20E6 recognizes the CD44 form containing variant exons v6–v10 but not the CD44v7–v10 form nor the standard CD44 molecule. Thus, it appears to specifically recognize an epitope encoded by the variant exon v6. 3G6 was used as a negative control tissues, the sections were run through a series of decreasingly graded ethanol baths. Immunoreactivity was triggered by a pre-treatment in acidic conditions (pH = 6.0) in a microwave oven. The Vectastain ABC kit (Vector laboratories, Inc, Burlingame, California) was used for the actual staining. Non-specific binding was blocked by normal horse serum. The primary antibodies were diluted in TBS (tris-buffered saline) containing 1% bovine serum albumin (BSA). Hermes-3 and 3G6 were supernatants from serum-free cultures and were used as 1:80 and 1:30 dilutions, respectively. 20E6 was an antibody concentrate used as a 10 $\mu g\ ml^{-1}$ dilution. The incubation with the primary antibody was done overnight at +4˚C. Biotinylated monoclonal IgG was used as the second stage reagent. The signal amplification was obtained by incubating the sections with avidin and biotinylated horseradish peroxidase for 30 minutes at room temperature. All of the incubations with antibodies and other reagents took place in humidified chambers at room temperature unless otherwise mentioned. Each of the above steps was followed by 3 washes in TBS (5 minutes each). 3,3-diaminobenzidine in TBS containing 0.03% $H_2O_2$ was used as a substrate for the peroxidase-mediated reaction, and Mayer's haematoxylin was used for the background staining. Finally, the sections were dehydrated in an increasingly graded series of ethanol, cleared in xylene and permanently mounted in DePex (BDH Limited, Poole, Dorset, UK). The antibody (Hermes-3, 20E6, 3G6) staining intensity was scored on a visual scale as – meaning no staining, + for weak staining, ++ corresponding to moderate staining and +++ for strong antibody binding. The staining intensities of lymphocytes as well as of epithelia were used as internal controls when determining the CD44 expression levels. In CD44v6 stainings epithelial staining intensity functioned as an internal control. The expression intensities were assessed by 3 independent experienced readers (SM, RR, PL) without the knowledge of any clinical data. Normal non-malignant tissues ($n$ = 9) representing the same tissue types from which the STS included in this study came from were also stained with the same antibodies and the general expression pattern of CD44 and CD44v6 was compared between normal and malignant tissues.

### Statistical analysis

The SPSS Macintosh computer program was used for the statistical analyses. The testing of the association between CD44/CD44v6 expression and proliferation, grade and size was done with Pearson's correlation coefficient test for continuous data and the Chi-square test for trends for categorial variables. The association between CD44/CD44v6 expression and local recurrence, distant recurrence and disease-specific survival was done with the Cox regression analysis with CD44/CD44v6 expression coded from 0 (negative) to 3 (+++). Risk ratios for local recurrence, distant recurrence and death were estimated from the same Cox model. Dermatofibrosarcomas (DFSP, $n$ = 8) were excluded from the overall survival and metastasis-free survival analyses as it is well known that these malignancies do not form distant recurrences.

### RESULTS

### CD44 expression

127 (95%) of the primary STS expressed CD44 and CD44v6 was detected in 76 (57%) of the neoplasms. Only 5 tumours (4%) entirely lacked CD44 expression. 45% of the tumours expressed CD44 as strongly as the normal epithelium (+++), 46% moderately (++) and the rest 9% revealed weak positivity (+) or no expression (–). In general, CD44v6 expression was relatively weak compared to that of all CD44; none of the samples stained strongly (+++) with the mAb 20E6, 9% showed moderate (++) staining, 50% stained faintly (+) and 43% were completely negative. The expression of both, CD44 in general as well as that of CD44v6 was mainly restricted to the cell membrane. CD44 and CD44v6

 © 2001 Cancer Research Campaign

**Table 2** The expression of CD44 and CD44v6 in 133 adult soft tissue sarcoma primary tumours

| Histotype | | CD44 Expression | | | | | | | | CD44v6 Expression | | | | | |
|---|---|---|---|---|---|---|---|---|---|---|---|---|---|---|---|
| | | – | | + | | ++ | | +++ | | – | | + | | ++ | |
| | *n* | *n* | (%) | *n* | (%) | *n* | (%) | *n* | (%) | *n* | (%) | *n* | (%) | *n* | (%) |
| MFH | 37 | 0 | (0) | 1 | (3) | 19 | (51) | 17 | (46) | 12 | (33) | 23 | (62) | 2 | (5) |
| LS | 15 | 0 | (0) | 0 | (0) | 10 | (67) | 5 | (33) | 6 | (40) | 7 | (47) | 2 | (13) |
| LMS | 14 | 0 | (0) | 1 | (7) | 5 | (36) | 8 | (57) | 5 | (36) | 9 | (64) | 0 | (0) |
| SS | 8 | 0 | (0) | 0 | (0) | 2 | (25) | 6 | (75) | 2 | (25) | 5 | (63) | 1 | (12) |
| FS | 8 | 0 | (0) | 0 | (0) | 3 | (37) | 5 | (63) | 5 | (63) | 1 | (12) | 2 | (25) |
| DFSP | 8 | 0 | (0) | 0 | (0) | 2 | (25) | 6 | (75) | 4 | (50) | 4 | (50) | 0 | (0) |
| ESEwing | 7 | 4 | (57) | 1 | (14) | 2 | (29) | 0 | (0) | 5 | (72) | 1 | (14) | 1 | (14) |
| MS | 4 | 0 | (0) | 0 | (0) | 3 | (75) | 1 | (25) | 2 | (50) | 2 | (50) | 0 | (0) |
| ESO | 2 | 0 | (0) | 0 | (0) | 1 | (50) | 1 | (50) | 0 | (0) | 2 | (100) | 0 | (0) |
| Epitheloid | 2 | 0 | (0) | 0 | (0) | 2 | (100) | 0 | (0) | 1 | (50) | 1 | (50) | 0 | (0) |
| Angiosarcoma | 2 | 0 | (0) | 0 | (0) | 1 | (50) | 1 | (50) | 0 | (0) | 2 | (100) | 0 | (0) |
| Other | 7 | 1 | (14) | 1 | (14) | 2 | (29) | 3 | (43) | 2 | (29) | 4 | (57) | 1 | (14) |
| Unclassified | 19 | 1 | (5) | 2 | (11) | 9 | (47) | 7 | (37) | 13 | (68) | 6 | (32) | 0 | (0) |

Abbreviations: MFH, malignant fibrous histiocytoma; LS, liposarcoma; LMS, leiomyosarcoma; ESEwing, extraskeletal Ewing's sarcoma; SS, synovial sarcoma; FS, fibrosarcoma; DFSP, dermatofibrosarcoma protuberans; MS, malignant schwannoma; ESO, extraskeletal osteosarcoma; Epithelioid, epithelioid sarcoma; Other, other sarcomas (see Table 1 for clarification).

© 2001 Cancer Research Campaign

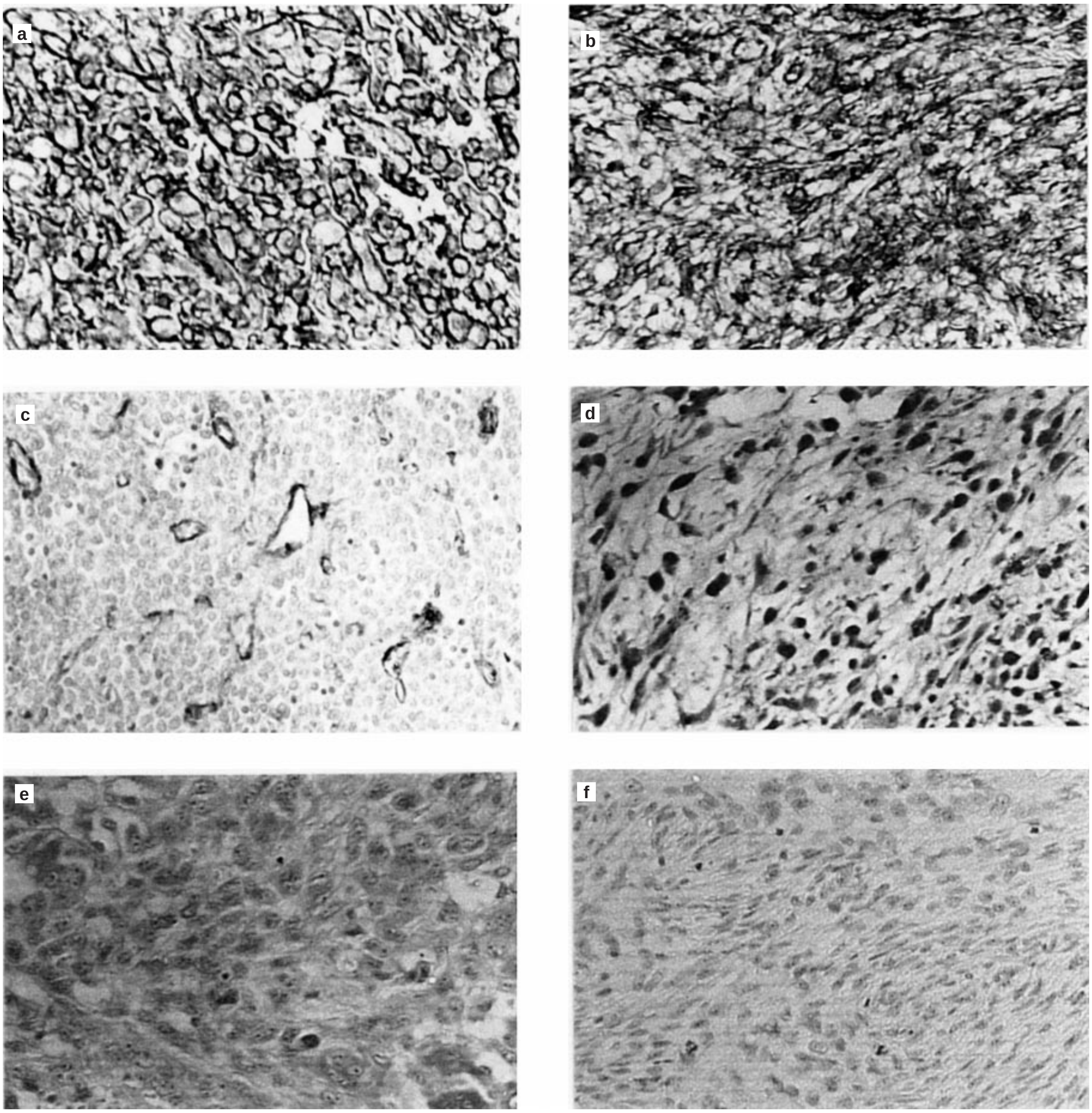

**Figure 2** Examples of the expression of CD44 (a, b, c) and CD44v6 (d, e, f) in human soft tissue sarcomas, *a*, Strong expression (+++) of CD44 in a MFH tumour. *b*, Moderate expression (++) of CD44 seen in another malignant fibrous histiocytoma. *c*, This is an unclassified sarcoma having no CD44 expression (–) in the tumour cells. *d*, Moderate (++) expression of CD44v6 in a MFH. *e*, Weak (+) and mainly cytoplasmic variant 6 expression was found in another MFH tumour. *f*, A CD44v6-negative (–) dermatofibrosarcoma. × 200

expression patterns are shown in detail by each STS group in Table 2. Examples of different expression levels are shown in Figure 2.

Stainings of mesenchymal tissue samples obtained from STS-free patients revealed moderate CD44 expression, the only exception being striated muscle where no CD44 was found. Stronger staining was seen in the endothelium of capillaries and larger blood vessels where the CD44 expression level resembling that of circulating blood lymphocytes. All of these non-malignant mesenchymal tissue samples revealed no expression or only weak expression (+) of the variant molecule CD44v6.

Synovial sarcomas consist of two distinct cell types, epithelial and spindle cells. Epithelial cells are known to contain cytokeratin and therefore we confirmed the location of the cells of epithelial origin in our synovial sarcoma material by performing an anti-cytokeratin staining with the monoclonal antibody AE1/AE3. We also found that CD44 was predominantly located in the epithelial components of these neoplasms when the cytokeratin staining was compared to the CD44 staining.

### Correlations between the expression and the clinicopathological parameters

Strong CD44 expression was found to be associated with low histological grade in the primary STS ($P = 0.04$); 97% (31/32) of the low-grade (grades I and II) and 88% (82/93) of the high-grade (grades III and IV) tumours expressed CD44 moderately or strongly (Table 3). In contrast, CD44v6 expression did not correlate with the tumour grade ($P > 0.1$). Expression of CD44, but not

 *© 2001 Cancer Research Campaign*

**Table 3** CD44 expression and histological grade in 125 adult primary soft tissue sarcoma tumours

| | | CD44 staining intensity | | | |
|---|---|---|---|---|---|
| **Grade** | ***n*** | **–** | **+** | **++** | **+++** |
| I | 8 | 0 (0%) | 0 (0%) | 5 (62%) | 3 (38%) |
| II | 24 | 1 (4%) | 0 (0%) | 11 (46%) | 12 (50%) |
| III | 50 | 0 (0%) | 2 (4%) | 24 (48%) | 24 (48%) |
| IV | 43 | 5 (12%) | 4 (9%) | 19 (44%) | 15 (35%) |

**Table 4** Univariate and multivariate analysis for local recurrence

| | Univariate analysis | | Multivariate analysis | |
|---|---|---|---|---|
| **Factor** | ***P*** | **Relative risk (95% CI)** | ***P*** | **Relative risk (95% CI)** |
| CD44 | 0.01 | 2.04 (1.2–3.6) | 0.12 | 1.5 (0.89–2.66) |
| CD44v6 | 0.05 | 1.7 (1.0–2.8) | 0.13 | 1.5 (0.89–2.41) |
| Adequate local treatment | 0.0001 | 0.28 (0.15–0.53) | 0.0009 | 0.33 (0.17–0.63) |

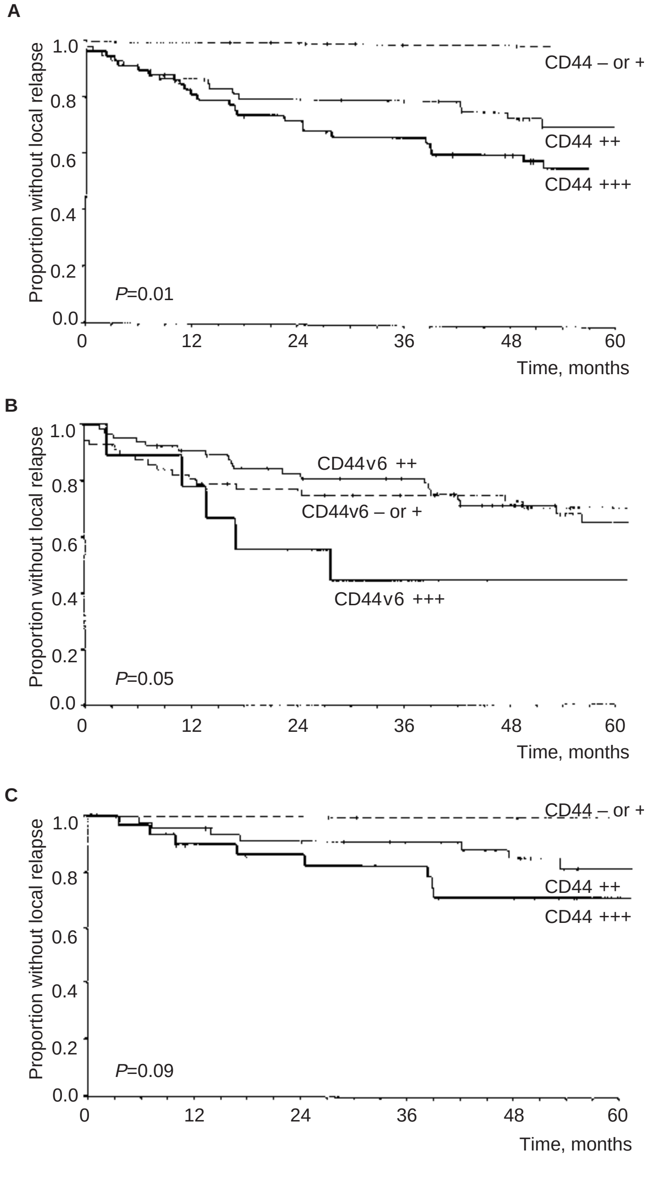

**Figure 3** (**A**) Local control in 133 adult patients with a STS by CD44 expression.(**B**) Local control in 133 adult patients with a STS by CD44v6 expression. (**C**) Local control in adequately treated patients with a STS ($n$ = 89) by CD44 expression that of the variant form v6, was also associated with the tumour size so that strong expression correlated with small size ($P$ = 0.02 and $P$ = 0.23). No significant statistical correlation was found between either CD44 or CD44v6 expression and the age at diagnosis, sex or tumour ploidy ($P$ > 0.1 for all the above comparisons).

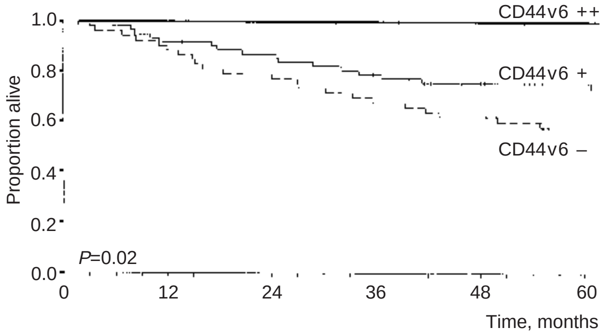

**Figure 4** Disease specific survival of 125 adult patients with a STS by CD44v6 expression

In the patient material as a whole, there was no overall correlation between CD44 expression and tumour proliferation rate assessed by S-phase fraction or Ki-67 expression ($P$ > 0.1 for all comparisons). In the group of diploid tumours ($n$ = 68), however, a significant inverse correlation between CD44 expression and the SPF was found (r = –0.40, $P$ = 0.001). A trend towards a correlation between CD44 and proliferation assessed by Ki-67 was also seen in diploid tumours (r = –0.23, $P$ = 0.09). No association could be found between the CD44v6 expression and the level of SPF or Ki-67 in diploid neoplasms ($P$ > 0.1 for both comparisons). Within the group of aneuploid tumours neither CD44v6 nor CD44 generally was significantly correlated with SPF or Ki-67 (SPF: $P$ = 0.72 for CD44 and $P$ = 0.45 for CD44v6; Ki-67: $P$ = 0.78 for CD44 and $P$ = 0.57 for CD44v6).

### Local recurrence

During the follow-up period 43 patients were seen to develop a local relapse. The factor most significantly associated with local recurrence was the adequacy of local treatment ($P$ = 0.0001), 63% ($n$ = 84) of the patients were considered as having received adequate local tumour treatment. Despite aggressive first line treatment 16 of these patients (19%) later had a local relapse. However, in the group of inadequately treated patients as many as 27 (55%) out of all 49 patients were later seen to develop a local recurrence. No significant correlation was seen when the incidence of local recurrences was compared with sex, histological tumour grade, size or SPF ($P$ > 0.1 for all comparisons).

Local relapses were seen more often in patients whose primary STS tumour expressed high levels of CD44 (Figure 3A, $P$ = 0.01) and also in patients having a STS with a high CD44v6 content (Figure 3B, $P$ = 0.05). In the group of adequately treated patients

© 2001 Cancer Research Campaign

**Table 5** Univariate and multivariate analysis for disease specific survival

| Factor | Univariate analysis | | Multivariate analysis | |
|---|---|---|---|---|
| | P | Relative risk (95% CI) | P | Relative risk (95% CI) |
| CD44 | 0.39 | 0.85 (0.6–1.2) | – | – |
| CD44v6 | 0.03 | 0.57 (0.3–1.0) | 0.13 | 0.65 (0.4–1.1) |
| Grade | 0.02 | 1.86 (1.2–2.8) | 0.03 | 1.58 (1.0–2.4) |
| Size | <0.00005 | 1.09 (1.0–1.1) | 0.0003 | 1.08 (1.0–1.1) |
| Adequate local treatment | 0.004 | 0.41 (0.2–0.8) | 0.0001 | 0.28 (0.1–0.5) |

there was a trend towards a higher local recurrence rate in tumours with high CD44 expression (risk ratio 2.2, $P = 0.09$) (Figure 3C) but no significant association between the CD44 variant v6 expression and local recurrence (risk ratio 1.78, $P = 0.65$). Within the patients having received non-optimal local treatment, on the other hand, a trend towards an association between the expression of CD44v6 and local recurrence was seen (risk ratio 1.78, $P = 0.06$) whereas no correlation was found between CD44 expression and local control rate (risk ratio 1.33, $P = 0.36$). In Table 4 we present the relative risks and 95% confidence intervals (95% CI) as well as $P$ values for the 3 factors, adequate local treatment, CD44 and CD44v6 expression in uni- and multivariate analyses concerning local recurrence.

### Survival

In univariate analysis low CD44v6 content of the primary STS tumour was found to predict poor prognosis ($P = 0.02$) (Figure 4). Adequate treatment, histological tumour grade and size of the neoplasm were other factors also associated with the survival and thus included in the multivariate analysis where they all seemed to be more independent prognostic indicators than CD44v6 expression. Results of the uni- and the multivariate analyses are presented in Table 5. The expression of CD44 seen in the primary STS tumour did not show any correlation with the survival ($P > 0.1$). Neither of the studied CD44 isoforms had any predictive value for metastasis-free survival ($P > 0.1$ for both analyses).

### DISCUSSION

The most interesting finding in this work was the more frequent incidence of local relapses in those STS patients whose tumour expressed a significant amount of CD44 or even more specifically the variant form containing the exon v6 protein domain. The risk was two-fold when compared between two expression level groups. Moreover, none of the strongly CD44-positive tumours that had been treated adequately according to common standards had metastasized. This finding may suggest that CD44 could have an anchoring role by tightly binding the primary tumour cells to the original tumour site. Tumour cells strongly expressing CD44 may have wider contacts with the surrounding cells, the ECM and growth-regulating cytokines. They could thereby possess better proliferation capacity and thus enhance tumour formation locally. On the other hand, as the malignant cells are tightly bound together as well as to the surrounding tissue they are less likely to detach from the tumour mass to form distant recurrences. This kind of behaviour of the CD44 molecule could also explain why, despite fairly benign grading and adequate primary treatment, certain tumours tend to form local recurrences.

In the present study, almost all primary soft tissue sarcoma tumours expressed CD44 molecules on their cell surfaces, at least to some extent, whereas the splice variant CD44v6 was found only in half of them. Fast proliferating tumours expressed only marginal levels of CD44 which also is consistent with the hypothesis of the CD44-molecule participating in local tumour control. Furthermore, high CD44 expression was associated with a low histological grade and small tumour size. Only 5 tumours were completely CD44-negative. All but one of them were extraskeletal Ewing's sarcomas. Ewing's sarcomas are primitive tumours that supposedly arise from early neuroectodermal cells. Picker et al have earlier shown that neurons generated from the same origin are CD44-negative, whereas glial cells expressed variable levels of CD44 (Picker et al, 1989). Our data concerning CD44 expression in Ewing's sarcomas, when the tumour origin is also considered, is thus congruent with these previous findings.

The structural details of CD44 are relatively well known but the functional relevance of the different CD44 isoforms is still poorly understood. The adhesive properties of CD44 seem to vary between different cell types and are affected by the activation status of the CD44 expressing cell. Despite controversial results, different CD44 isoforms seem to have differential ligand binding properties (Jackson et al, 1995). CD44, and especially its splice variants, have been shown to have a critical role in embryogenesis directing growth and differentiation of developing cells (Ruiz et al, 1995) and this physiological function is generally thought to closely resemble malignant growth. Certain cytokines are known to modify the function of this adhesion molecule group, perhaps leading to changes in the cytoskeletal organization of a cell and in cell motility which, again, are also necessary features for metastasizing tumour cells.

CD44v6 was earlier thought to be an adhesion molecule universally related to metastasis formation but the more different kinds of tumour groups have been studied the more complicated its role has become. A number of studies emphasizing the essential role of CD44v6 in tumour cell spreading have been published (Koopman et al, 1993; Herrlich et al, 1995; Kainz et al, 1995; Mulder et al, 1995), however, it has become obvious that CD44v6 behaves differently in different neoplastic groups; depending on the tumour type both increases and decreases of the cell surface CD44v6 expression have been correlated with the disease-specific outcome (Naor et al, 1997). Poor survival related with low CD44v6 expression has been mainly reported in squamocellular carcinomas and in transitional cell carcinomas of the bladder (Hong et al, 1995; Hudson et al, 1996; Ross et al, 1996; Seelentag et al, 1996; Sugino et al, 1996). Similarly, in the case of STS, lack rather than excess of CD44v6 was correlated with poor survival and hence CD44v6 could be suitable as a useful prognostic factor in these malignancies. Perhaps loose interaction with the surrounding cells and the extracellular matrix enhance tumour cell detachment from their environment, tumour cell migration and haematogenic metastasis formation. This hypothesis is supported not only by our results concerning local recurrences and the survival, but also by the data presented by Soukka et al where they showed with squamocellular carcinomas that diminished CD44v6-expression was associated with poorly differentiated rapidly growing neoplasms (Soukka et al, 1997). In a very recent report by Ishida, a decreased CD44v6 expression was associated with a higher probability of invasion but not of metastasis formation in colorectal adenocarcinomas further supporting our finding (Ishida, 2000).

 © 2001 Cancer Research Campaign

Recently, Kahara et al published a small descriptive study on CD44 expression in 47 STS. They found that both CD44v6 and CD44v9 were the predominately expressed CD44 variants in STS validating our study. Despite the restricted patient material they found a correlation between CD44v6 expression and metastasis-free survival (Kahara et al, 2000). That could not be found in our study, perhaps because of larger histological heterogeneity in our tumour material. The same reason may also, at least partially, explain why we found a correlation between low CD44v6 expression and the survival in univariate analysis but failed to confirm the result in multivariate analysis.

In conclusion, this was the first time the expression and clinical importance of CD44, an adhesion molecule family often linked with tumour prognosis, was studied in STS. We found both the standard and the variant form v6 of CD44 molecule commonly expressed in STS. The expression of CD44 was related to the incidence of local recurrences as well as to tumour size and grade. Expression of CD44v6 was also linked to the later occurrence of local relapses. Moreover, low expression of CD44v6 predicted poor disease-specific survival. These findings, if confirmed, could be of great clinical relevance as the tumours with a high CD44 expression were associated with a higher risk for local failure and a lower risk for distant recurrence. They would thus be ideal candidates for determining the patients benefiting from intensified local treatment.

### ACKNOWLEDGEMENTS

We thank Professor Sirpa Jalkanen for providing us with the laboratory facilities and reagents. Paraffin embedded samples of healthy tissues were received as a kind gift from Dr Karl-Ove Söderström. The CD44 construct containing the exons v7–v10 was generously donated by Dr David Jackson. We also want to address our thanks to Mrs Marika Iljamo for teaching us immunohistochemical preparation and staining of the samples. Dr David Smith is thanked for the language revision and Anne Sovikoski-Georgieva for irreplaceable secretarial help.

### REFERENCES

Aho R, Kalimo H, Salmi M, Smith D and Jalkanen S (1997) Binding of malignant lymphoid cells to the white matter of the human central nervous system: Role of different CD44 isoforms, $\beta_1$, $\beta_2$ and $\beta_7$ integrins and L-selectin. *J Neuropathol Exp Neurol* **56**: 557–568

Alvegård TA, Berg NO, Ranstam J, Rydholm A and Rooser B (1989) Prognosis in high-grade soft tissue sarcomas. The Scandinavian Sarcoma Group experience in a randomized adjuvant chemotherapy trial. *Acta Orthop Scand* **60**: 517–521

Belitsos PC, Hilderth JEK and August JT (1990) Homotypic cell aggregation induced by anti-CD44 (Pgp-1) monoclonal antibodies and related to CD44 (Pgp-1) expression. *J Immunol* **144**: 1661–1670

Collin C, Godbold J, Hajdu S and Brennan M (1987) Localized extremity soft tissue sarcoma: an analysis of factors affecting survival. *J Clin Oncol* **5**: 601–612

Collin F, Chassevent A, Bonichon F, Bertrand G, Terrier P and Coindra JM (1997) Flow cytometric DNA content analysis of 185 soft tissue neoplasms indicates that S-phase fraction is a prognostic factor for sarcomas. French Federation of Cancer Centers (FNCLCC) Sarcoma Group. *Cancer* **79**: 2371–2379

Choong PF, Åkerman M, Willen H, Andersson C, Gustafson P, Alvegård T and Rydholm A (1995) Expression of proliferating cell nuclear antigen (PCNA) and Ki-67 in soft tissue sarcoma. Is prognostic significance histotype-specific? *APMIS* **103**: 797–805

Denning SM, Le PT, Singer KH and Haynes BF (1990) Antibodies against the CD44 p80, lymphocyte homing receptor molecule augment human peripheral blood T cell activation *J Immunol* **144**: 7–15

Dirix LY and Van Oosterom AT (1995) Diagnosis and treatment of soft tissue sarcomas in adults. *Curr Opin Oncol* **7**: 340–348

Günthert U, Hofmann M, Rudy W, Reber S, Zoller M, Haussmann I, Matzku S, Wenzel A, Ponta H and Herrlich P. (1991) A new variant of glycoprotein CD44 confers metastatic potential to rat carcinoma cells. *Cell* **65**: 13–24

Herrlich P, Pals S and Ponta H (1995) CD44 in colon cancer. *Eur J Cancer* **31A**: 1110–1112

Hong RL, Pu YS, Hsieh TS, Chu JS and Lee WJ (1995) Expressions of E-cadherin and exon v6-containing isoforms of CD44 and their prognostic values in human transitional cell carcinoma. *J Urol* **153**: 2025–2028

Hudson DL, Speight, PM and Watt FM (1996) Altered expression of CD44 isoforms in squamous-cell carcinomas and cell lines derived from them. *Int J Cancer* **66**: 457–463

Huuhtanen RL, Blomqvist CP, Wiklund TA, Virolainen MJ, Elomaa AI, Pan Y and Tribukait B (1996) S-phase fraction of 155 soft tissue sarcomas: correlation with clinical outcome. *Cancer* **77**: 1815–1822

Huuhtanen RL, Blomqvist CP, Wiklund TA, Böhling TO, Virolainen MJ, Tukiainen EJ, Tribukait B and Andersson LC (1999) Comparison of the Ki-67 score and S-phase fraction as prognostic variables in soft-tissue sarcoma. *Br J Cancer* **79**: 945–951

Ishida T (2000) Immunohistochemical expression of the CD44 variant 6 in colorectal adenocarcinoma. *Surg Today* **30 (1)**: 28–32

Jacobson K, O'Dell D, Holifield B, Murphy TL and August JT (1984) Redistribution of a major cell surface glycoprotein during cell movement. *J Cell Biol* **99**: 1613–1623

Jackson DG, Bell JI, Dickinson R, eTimans J, Shields J and Whittle N (1995) Proteoglycan forms of the lymphocyte homing receptor CD44 are alternatively spliced variants containing the v3 exon. *J Cell Biol* **128**: 673–685

Jalkanen S, Bargatze RF, Herron LR and Butcher EC (1986) A lymphoid cell surface glycoprotein involved in endothelial cell recognition and lymphocyte homing in man. *Eur J Immunol* **16**: 1195–1202

Jalkanen S, Bargatze RF, de los Toyos J and Butcher EC (1987) Lymphocyte recognition of high endothelium: antibodies to distinct epitopes of an 85–95-kD glycoprotein antigen differentially inhibit lymphocyte binding to lymph node, mucosal, or synovial endothelial cells. *J Cell Biol* **105**: 983–990

Kahana N, Ozaki T, Doi T, Nishida K, Kawai A, Shibahara M and Inoue H (2000) CD44 expression in soft tissue sarcomas. *Virchows Arch* **436**: 574–578

Kainz C, Kohlberger P, Tempfer C, Sliutz G, Gitsch G, Reinthaller A and Breitenecker G (1995) Prognostic value of CD44 splice variants in human stage III cervical cancer. *Eur J Cancer* **31A**: 1706–1709

Koopman G, Heider KH, Horst E, Adolf GR, van den Berg F, Ponta H, Herrlich P and Pals ST (1993) Activated human lymphocytes and aggressive non-Hodgkin's lymphomas express a homologue of the rat metastasis – associated variant of CD44. *J Exp Med* **177**: 897–904

Mackay CR, Terpe HJ, Stauder R, Marston WL, Stark H and Günthert U (1994) Expression and modulation of CD44 variant isoforms in humans. *J Cell Biol* **124**: 71–82

Miyake K, Underhill CB, Lesley J and Kincade PW (1990a) Hyaluronate can function as a cell adhesion molecule and CD44 participates in hyaluronate recognition. *J Exp Med* **172**: 69–75

Miyake K, Medina KL, Hayashi S-I, Ono S, Hamaoka T and Kincade PW (1990b) Monoclonal antibodies to Pgp-1/CD44 block lymphohematopoiesis in long-term bone marrow cultures. *J Exp Med* **171**: 477–488

Mulder JWR, Wielenga VJM, Polak MM, van den Bergh FM, Adolf GR, Herrlich P, Pals ST and Offerhaus GJ (1995) Expression of mutant p53 and CD44 variant proteins in colorectal tumorigenesis. *Gut* **36**: 76–80

Naor D, Sionov R and Ish Shalom D (1997) CD44: structure, function, and association with the malignant process. *Adv Cancer Res* **71**: 241–319

Picker LJ, Nakache M and Butcher EC (1989) Monoclonal antibodies to human lymphocyte homing receptors define a novel class of adhesion molecules on diverse cell types. *J Cell Biol* **109**: 927–937

Ross JS, del Rosario AD, Bui HX, Kallakury BV, Okby NT and Figge J (1996) Expression of the CD44 cell adhesion molecule in urinary bladder transitional cell carcinoma. *Mod Pathol* **9**: 854–860

Ruiz P, Schwarzler C and Günthert U (1995) CD44 isoforms during differentiation and development. *Bioessays* **17**: 17–24

Rööser B, Attewell R, Berg NO and Rydholm A (1987) Survival in soft tissue sarcoma. Prognostic variables identified by multivariate analysis. *Acta Orthop Scand* **58**: 516–522

Seelentag WK, Günthert U, Saremaslani P, Futo E, Pfaltz M, Heitz PU and Roth J (1996) CD44 standard and variant isoform expression in human epidermal skin tumors is not correlated with tumor aggressiveness but down-regulated during proliferation and tumor de-differentiation. *Int J Cancer* **69**: 457–463

*© 2001 Cancer Research Campaign*

Soukka T, Salmi M, Joensuu H, Häkkinen L, Sointu P, Koulu L, Kalimo K, Klemi P, Grenman R and Jalkanen S (1997) Regulation of CD44v6-containing isoforms during proliferation of normal and malignant epithelial cells. *Cancer Res* **57**: 2281–2289
Sugino T, Gorham H, Yoshida K, Bolodeoku J, Nargund V, Cranston D, Goodison S and Tarin D (1996) Progressive loss of CD44 gene expression in invasive bladder cancer. *Am J Pathol* **149**: 873–882
Taubert H, Meye A and Wurl P (1996) Prognosis is correlated with p53 mutation type for soft tissue sarcoma patients. *Cancer Res* **56**: 4134–4136
Webb DSA, Shimizu Y, van Seventer GA, Shaw S and Gerrard TL (1990) LFA-3, CD44, and CD45: physiologic triggers of human monocyte TNF and IL-1 release. *Science* **249**: 1295–1297
Wiklund T, Huuhtanen R, Blomqvist C, Tukiainen E, Virolainen M, Virkkunen P, Asko-Seljavaara S, Björkenheim JM and Elomaa I (1996) The importance of a multidisciplinary group in the treatment of soft tissue sarcomas. *Eur J Cancer* **32A**: 269-273

 *© 2001 Cancer Research Campaign*
